# Scatter rejection and scatter correction for single and multiple exposure static diagnostic CT

**DOI:** 10.1002/mp.70749

**Published:** 2026-11-11

**Authors:** Andreas Heinkele, Julien Erath, Lukas Hennemann, Johan Sunnegårdh, Christian Hofmann, Martin Petersilka, Karl Stierstorfer, Marc Kachelrieß

**Affiliations:** ^1^ Computed Tomography Division Siemens Healthineers AG Forchheim Germany; ^2^ Division of X‐ray Imaging and CT German Cancer Research Center (DKFZ) Heidelberg Germany; ^3^ Medical Faculty Heidelberg Heidelberg University Heidelberg Germany

**Keywords:** scatter correction, static CT, stationary CT

## Abstract

**Background:**

Static CT designs may offer several advantages, including increased compactness and mechanical simplification. In addition, acquisition speed is no longer constrained by gantry rotation but instead determined by electronic source switching and source power. Static CT further enables multiple simultaneous exposures, for example, in dual‐source or even triple‐source mode. Due to the static geometry, conventional anti‐scatter grids (ASGs) cannot be employed, making scattered radiation a major challenge.

**Purpose:**

To investigate how 4th generation geometry static CTs can be made suitable for general purpose diagnostic CT imaging with regard to their scattered radiation characteristics.

**Methods:**

In this work, we investigate how scatter can be addressed, for example, by using defocused ASGs for scatter rejection and scatter correction approaches. Our evaluation focuses not only on the correction of scatter‐induced artifacts but also on the overall image quality, particularly with respect to noise degradation caused by scattered radiation. To do so, we simulate a 4th generation geometry static CT with different levels of collimation in single‐source and triple‐source exposure mode. It is equipped with a 360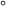 detector ring and a 360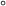 source ring array, eliminating the need for rotating components. For scatter rejection, we investigate the potential benefits of one‐dimensional ASGs in the *z*‐direction by comparing image quality across different ASG heights and phantom sizes. For scatter correction, we employ the deep scatter estimation (DSE), which is based on a U‐Net architecture and trained with Monte Carlo–generated scatter estimates. Performance is evaluated with respect to residual scatter artifacts and noise amplification.

**Results:**

DSE effectively corrects scatter artifacts in both single‐source and multi‐source operation, yielding reconstructions in which scatter artifacts are strongly reduced. However, in multi‐source operation we observe a notable increase in image noise at constant dose levels. The use of a one‐dimensional ASG in the *z*‐direction can mitigate this noise increase and thereby enable dose savings.

**Conclusions:**

Deep learning‐based methods such as DSE can effectively address scatter artifacts. However, the absence of an ASG results in increased image noise. Incorporating a one‐dimensional ASG in the z‐direction can reduce noise and thereby may enable dose savings.

## 1 INTRODUCTION

### 1.1 Background

Static CT can be considered an advancement of 4th generation scanners, which were originally rotate‐stationary systems.^1^ These scanners retained a rotating source similar to 3rd generation scanners, but differed by incorporating a stationary detector ring. One exception is the electron‐beam‐CT (EBT), which uses a static electron source and sweeps the focal spot along an anode by electromagnetic deflection of the electron beam.^2^ The first 4th generation scanners were introduced in 1977.^1^ This geometry was developed to address challenges encountered with rotate‐rotate scanners,^3^ such as ring artifacts caused by rotating detector elements and motion artifacts due to cardiac and respiratory movement. 4th generation scanners inherently avoided ring artifacts while achieving comparable temporal resolution to 3rd generation scanners. However, this advantage came at the cost of increased expenses, as more detector elements are required to assemble a complete detector ring. Moreover, 4th generation scanners inherently disallow the use of anti‐scatter grids (ASGs) because the lamellae of an ASG at one detector element cannot follow the rotating source. This limitation led to significant scatter artifacts, and available scatter correction methods did not achieve sufficient accuracy. Combined with improvements in acquisition speed and ring artifact correction in 3rd generation CT, these factors explain why 4th generation scanners did not succeed commercially in the long term. Although nearly all clinical scanners today are 3rd generation systems, research has explored various alternative geometries, including inverse‐geometry CT,^4^ multi‐source CT,^5^, ^6^, ^7^ and multi‐source static CT.^8^ In this work, we simulate a static CT geometry as illustrated in Figure 1 with a 16 row detector ring, corresponding to a *z*‐coverage of approximately 12 mm at the isocenter.

**FIGURE 1:**
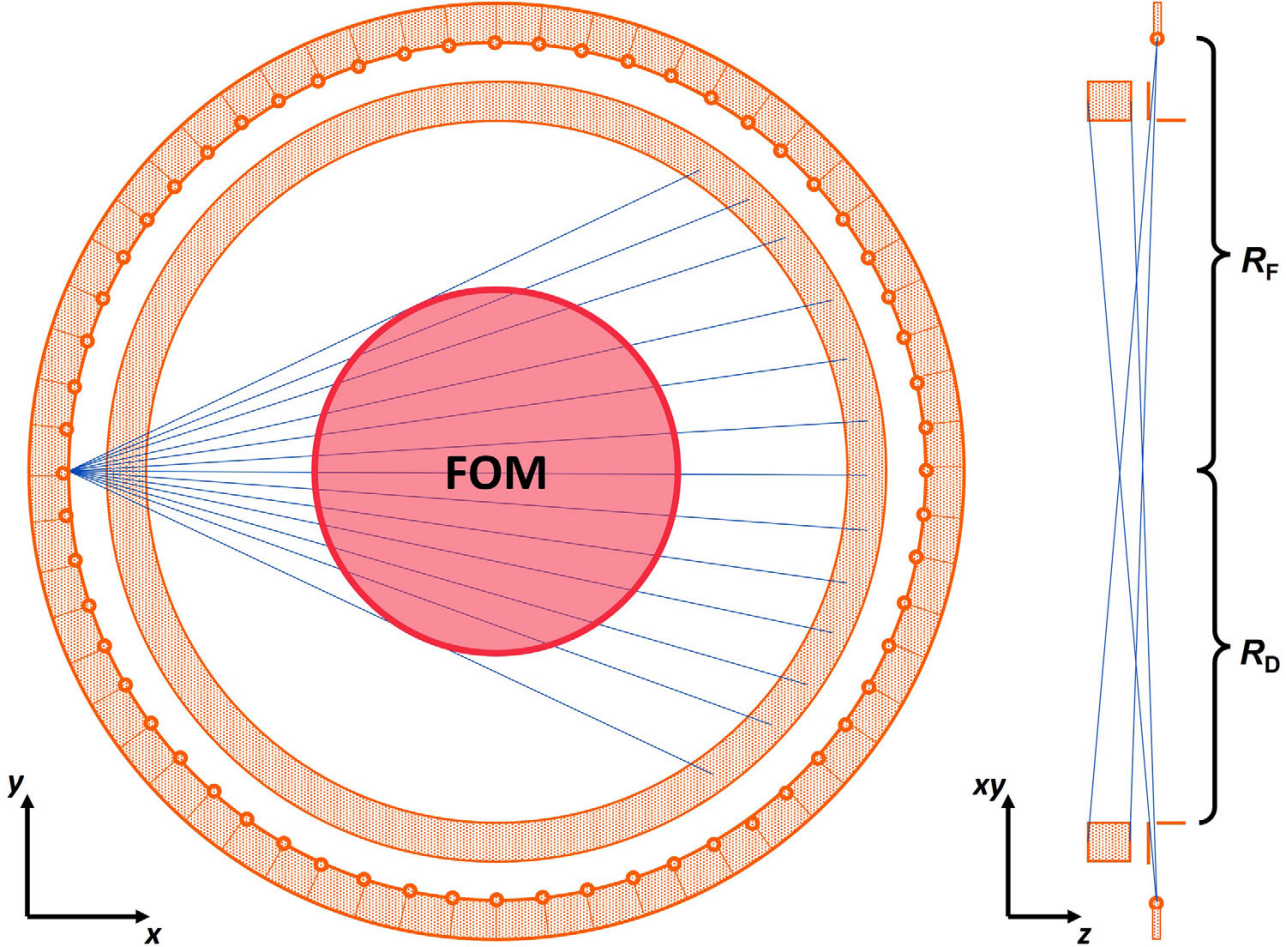
Static CT geometry, not drawn to scale. On the left, the geometry is shown in *x*‐*y*‐plane. The outer ring is the source array, the inner ring the detector. On the right, the geometry is shown in *y*‐*z*‐plane. Detector and source ring need to be offset in *z*‐direction. $R_{F}$ and $R_{D}$ denote the radius of the source ring and detector ring, respectively. The x‐ray beam is collimated using a ring‐shaped collimator.

Additionally to the static detector ring, the system is equipped with a static ring source array which consists of a large number of individual sources. These sources may be based on carbon nanotubes (CNTs). Unlike conventional x‐ray tubes, where electrons are extracted from the cathode by thermionic emission, the cathode of CNTs is not heated and electrons are extracted by field emission which allows high electronic switching speeds.^9^ The static CT geometry offers several advantages compared to 3rd generation scanners: The absence of a rotating gantry leads to an enormous mechanical simplification and allows a more compact design. This will also reduce cost for the mechanical assembly. Moreover, the acquisition time is not limited by the rotation speed of the gantry but rather by the electronic switching time and power of the x‐ray sources. A static CT system imposes less mechanical constraints for multi‐source operation compared to for example, dual‐source systems that are based on 3rd generation CT geometry. The number of simultaneously active sources is only limited by the field of measurement and the scanner geometry, i.e. radius of detector, source array and collimator, and *z*‐offset between source and detector. With a certain amount of active sources the primary fan beams will overlap and make the acquired data less suitable or even unusable. The rationale for simultaneously activating multiple sources is to enhance temporal resolution. Maximizing the temporal resolution is important for example, for cardiac imaging, since the images are prone to motion artifacts. In clinical CT today, dual‐source scanners (DCCT) are high‐end devices with acquisition times as low as 66 ms. The two sources in DCCT can either be used to obtain spectral information or to increase temporal resolution. Both options would be feasible with static CT. The compact design may make static CT attractive for some special or portable applications. These could include mobile stations, intensive care units, and interventional radiology. In addition, the prospect of fast acquisition times makes static CT particularly attractive for applications such as cardiac imaging, where reconstructions are highly susceptible to motion artifacts. The prospective high temporal resolution enabled by static CT is critical for accurately capturing rapidly moving structures, such as the coronary arteries, and may improve diagnostic confidence in detecting stenosis or other cardiovascular abnormalities. Beyond cardiology, improved temporal resolution could also benefit imaging of other dynamic processes, for example, in perfusion studies or interventional settings. However, state‐of‐the‐art CNT‐based sources have only limited power compared to conventional sources.^10^, ^11^ This increases the acquisition time for high‐dose images and limits possible advantages regarding temporal resolution to low‐dose applications. Technological advances might mitigate this issue in the future. Other drawbacks of static CT exist. About three to four times more detector elements are necessary to assemble an entire detector ring compared to a typical single‐source scanner with 50 cm field of measurement, causing additional costs. Beam collimation is performed using a ring collimator, as equipping each focal spot with an individual collimator is impractical given the large number of sources. As a result, the collimation is inherently non‐uniform since the distance between the focal spot and the collimator ring varies with the fan angle. Two implications are possible. First, to enable full detector usage within the field of measurement (FoM), the beam needs to be overcollimated in the projection center which causes a waste of dose. Second, undercollimation prevents waste of dose, but leads to low *z*‐coverage and a detector that is only partially illuminated. One of the biggest challenges, however, is scattered radiation since the scanner geometry inherently disallows the use of conventional ASGs. In this manuscript we want to discuss if this challenge can be overcome in the future.

### 1.2 Scatter correction

Monte Carlo (MC) simulations are considered the gold standard for scatter estimation. MC methods are based on physics models that account for all relevant interactions of x‐ray photons with matter. By propagating a large number of photons through the object of interest, highly accurate scatter estimates can be obtained. However, MC simulations are computationally expensive and therefore unsuitable for real‐time applications. Consequently, models with simplified physical assumptions are often used in clinical scanners.^12^, ^13^, ^14^ Such simplified models may follow a convolution‐based approach and can be combined with MC simulations to improve performance.^15^ While these models are computationally efficient, they lack accuracy. A large variety of scatter estimation models exist and the interested reader may be referred to the comprehensive summary provided by Rührnschopf et al.^16^, ^17^ In recent years, research has increasingly focused on deep learning‐based approaches.^18^, ^19^, ^20^, ^21^, ^22^ Recent other work that focuses on scatter correction for static CT has been published by Xia et al.^23^, ^24^ They created a framework for static CT scatter correction with 5‐source operation for application in cardiac imaging. Scatter‐corrected images reported in their work appear promising. However, image noise is expected to increase significantly when shooting with five sources due to the quantum noise caused by scatter, thereby compromising image quality and increasing the required dose and potentially the acquisition time to achieve adequate image quality. McSkimming et al. published an adaptive deep scatter estimation for multi‐source stationary or semi‐stationary cone‐beam CT for stroke imaging. They introduce an iterative framework that uses a U‐net for scatter estimation in a surrogate geometry.^25^ Deep scatter estimation (DSE), introduced in 2018,^18^ is based on a U‐net architecture and trained to estimate the scattered signal from acquired projection data. DSE is typically trained on MC‐simulated data. However, MC‐free DSE implementations are also possible.^26^ Previous research has shown that DSE performance is robust to variations in tube voltage and noise level, provided these variations are represented in the training data.^27^ A DSE model trained across diverse parameter settings can therefore be applied to a range of acquisition protocols. DSE remains an active topic of research with potential applications across various CT systems. For example, DSE has been applied to cone‐beam CT,^27^ dual‐source CT where cross‐scatter must also be estimated,^28^ and photon‐counting CT.^29^, ^30^ In this work, we apply DSE to a static CT geometry. Since DSE has been extensively investigated for 3rd generation scanners, we first present single‐source simulations as a proof of concept study, demonstrating that DSE can also be applied to static CT geometries. Subsequently, DSE is employed to correct scatter in dual‐source and triple‐source modes. One drawback of purely software‐based scatter correction is that it can only compensate for the expected value of the scatter, but not for the associated quantum noise. Consequently, scatter‐corrected images exhibit increased noise.^31^ In this work, we aim to quantify the extend of noise increase for single‐ and multi‐source operation and to investigate implications, such as the impact on the required dose and the conditions under which multi‐source operation may be beneficial. In addition to software‐based correction, 3rd generation scanners typically employ ASGs to physically reject scattered radiation, thereby reducing artifacts and image noise. Since conventional ASGs cannot be used in static CT, we propose the use of one‐dimensional ASGs. Besides demonstrating the feasibility of deep learning‐based scatter correction for static CT, we conduct a proof‐of‐concept study to investigate how such ASGs affect image noise and the required dose, and to determine under which conditions static CT may benefit from their use.

## 2 METHODS

### 2.1 Dataset generation

Two datasets were created: one for training and validation of DSE, and another for testing. For training and validation, we generated a paired dataset of scatter‐contaminated projection data and corresponding scatter‐only data using our in‐house MC simulation software.^32^, ^33^ The dataset consists of circular and elliptical water phantoms and the FORBILD^34^ thorax phantom. We simulated circular scans with an angular offset of 5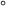 between projections. For each circular scan, the object size and lateral position, as well as the *z*‐position of the thorax phantom and the aspect ratio of the elliptical phantom were varied according to Table 1.

**TABLE 1:** Composition of the training dataset.

	**Cylindrical water**	**Elliptical water**	**FORBILD thorax**
Number of scans	50	50	100
*x*‐shift	0 cm	0–2 cm	0–8 cm
*y*‐shift	0–8 cm	0–5 cm	0–8 cm
Size	8–20 cm radius	7.5–15 cm and 15–25 cm half axes	0.5–1.3 scaling

In total, 50 circular scans of circular water phantoms, 50 circular scans of elliptical water phantoms and 100 circular scans of the thorax phantom were acquired, resulting in a total number of 200 scans with 72 projections per scan. This yields a dataset with 14  400 projections. The training / validation split of the dataset is 90 % / 10 %. The phantoms used for training are visualized in Figure 2.

**FIGURE 2:**
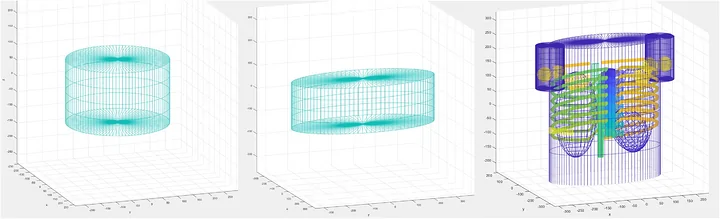
Surface representation of the phantoms that are used for DSE training. From left to right: circular water phantom, elliptical water phantom, semi‐anthropomorphic thorax phantom. For dataset generation, the phantoms were varied in size, lateral shift, aspect ratio (elliptical phantom) and *z*‐position (thorax).

For the generation of the test dataset, we used three phantoms: The FORBILD^34^ head phantom, a XCAT^35^, ^36^, ^37^ abdomen phantom resembling a realistic patient anatomy, and a XCAT chest phantom with contrast agent, where the CT value of blood was set to 300 HU.

Specifications of the static CT scatter simulations are summarized in Figure 2.

**TABLE 2:** (A) MC simulation parameters and characteristics and (B) Static CT system specifications.

(A) MC parameters and characteristics	
Code & version	MOCASSIM (Siemens Healthineers), v10.41
Validation	Stierstorfer et al.^32^, ^33^
Tube voltage	120 kV
Spectrum	Measured, with additional 2 mm aluminum filter
Detector	Energy‐integrating, ideal detector response
Interaction models	Photo absorption, Compton, Rayleigh, K‐emission
Cross‐sections	EADL and EPDL (NEA/LLNL libraries)
**(B) Static CT system specifications**	
Radius of source ring	600 mm
Radius of detector ring	450 mm
*z*‐coverage (isocenter)	12 mm
Bowtie filter	None
Additional prefilter	None
Acquisition mode	Circle scan

Simulations were performed with a tube voltage of 120 kV. Beam‐shaping filters, such as bowtie filters, were omitted, since their implementation would be challenging given the large number of x‐ray sources and the associated geometric constraints. No additional pre‐filtration was applied in the presented studies, although the use of ring‐shaped filters would, in principle, be possible. The employed x‐ray spectrum already includes an inherent filtration equivalent to 2 mm of aluminum. The geometry, that is, the detector and source radii, was chosen to enable diagnostic CT imaging and thus corresponds to the typical configurations of fourth generation CT scanners used in the 1980s and 1990s. Systems with more unconventional arrangements of source and detector arrays, that may be interesting for specialized imaging applications, are not general purpose radiological imaging devices and therefore not within the scope of our paper and thus not discussed.

### 2.2 Deep scatter estimation (DSE)

DSE is based on a U‐net architecture and is trained on MC‐simulated data. An overview of the architecture is illustrated in Figure 3.

**FIGURE 3:**
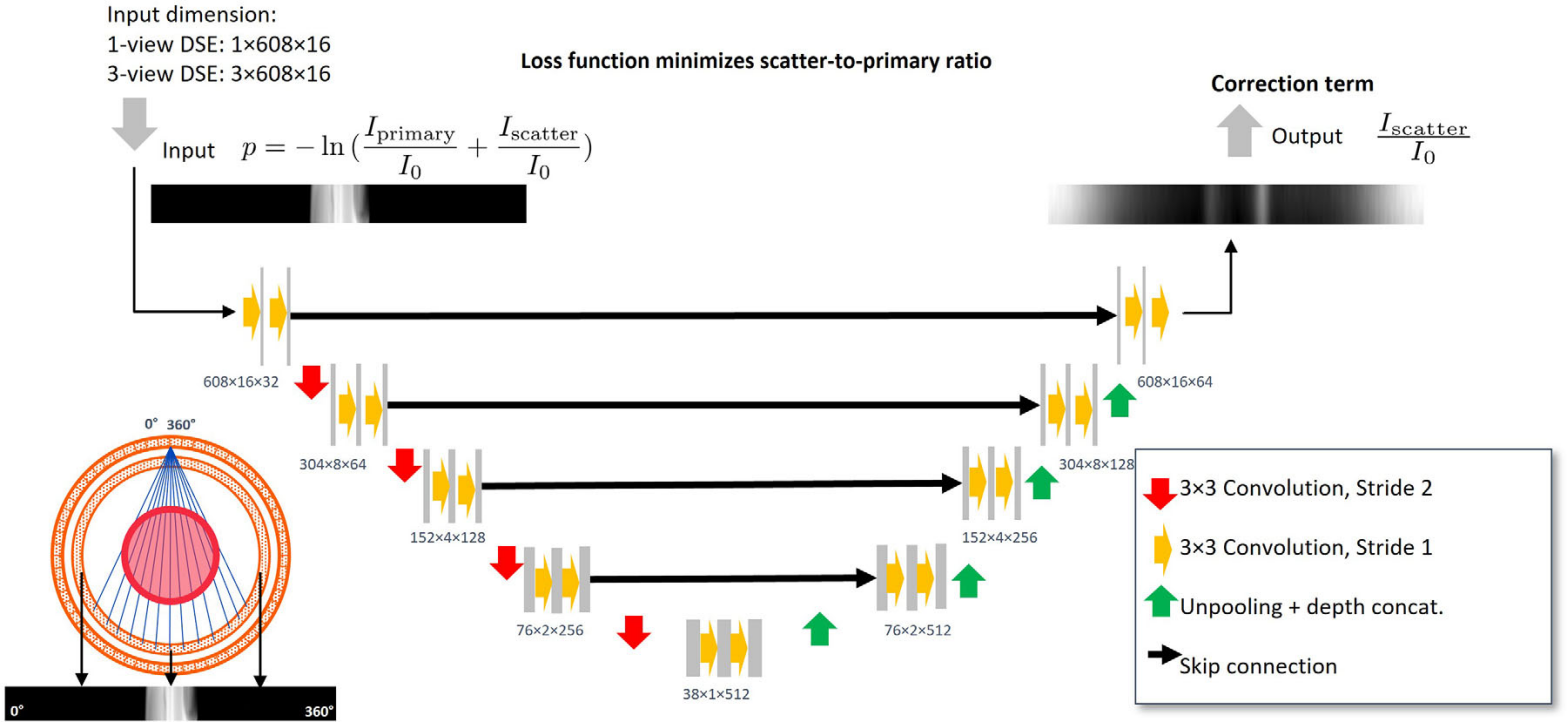
DSE is based on a U‐net architecture. It is trained to estimate the scatter distribution from the logarithmic projection data. As a loss function, we use the scatter‐to‐primary mean absolute percentage error (SPMAPE), limited to a 50 cm field of measurement.

Input to the DSE is the scatter‐contaminated logarithmic projection data. Since static CT is equipped with a full detector ring, the scatter measured outside the primary fan beam is also available to DSE. These additional data provide more spatial information, which may be particularly valuable for estimating cross‐scatter. The input was downsampled by a factor of four since we obtained more accurate estimates in terms of validation loss compared to applying it to the full‐sized projection. Output of DSE is the scatter‐only projection in intensity domain. After estimation with DSE, the output was upsampled again by linear interpolation. The upsampling does not impair prediction accuracy since the scatter distribution is a low‐frequency signal. The weights and biases of the U‐net were optimized by minimizing the scatter‐to‐primary mean absolute percentage error (SPMAPE). The SPMAPE was chosen as loss function since it was demonstrated that the scatter‐to‐primary ratio (SPR) correlates well with the scatter artifacts in the reconstructed image.^28^ The optimization is defined by (1):

(1) $$\text{SPMAPE}=\frac{1}{N}\sum \left|\frac{I_{\text{scatter,MC}}-I_{\text{scatter,DSE}}}{I_{\text{prim}}}\right|$$

where $I_{\text{scatter,MC}}$ and $I_{\text{scatter,DSE}}$ denote the scatter signals obtained by MC simulation and DSE prediction, respectively, and with N being the number of detector pixels. Table 3 summarizes specifications and hyperparameters of DSE, which were heuristically chosen to minimize the validation loss.

**TABLE 3:** Configuration for DSE training.

Batch size	8
Epochs	500
Learning rate	$10^{-5}$
Learning rate reduction if validation loss does not improve	Factor 2 after 100 epochs
Filters in first layer	32
Loss function	SPMAPE
Optimizer	Adam
Activation	ReLU
Input downsampling factor	4

After applying DSE, scatter correction is done by subtracting the DSE‐estimate from the total projection data. For single‐source simulations, we subtract the estimate according to:

$$\begin{array}{c} I_{\mathrm{prim}}=I_{\mathrm{total}}-I_{\mathrm{scatter}},\mathrm{DSE}. \end{array}$$

For multi‐source simulations where very high SPRs are expected, scatter subtraction is performed as follows:

$$\begin{array}{cc} -\ln I_{\mathrm{prim}} & =-\ln(I_{\mathrm{total}}-I_{\mathrm{scatter}})\\ & =-\ln I_{\mathrm{total}}-\ln\left(1-\frac{I_{\mathrm{scatter}}}{I_{\mathrm{total}}}\right)\\ & \approx -\ln I_{\mathrm{total}}-\ln\left(1-\frac{I_{\mathrm{scatter}},\mathrm{DSE}}{I_{\mathrm{total}}}\right). \end{array}$$

To avoid overcorrection, the second term is approximated by a Taylor series as previously shown in Petersilka et al.^38^:

$$\begin{array}{c} \ln\left(1-\frac{I_{\mathrm{scatter}},\mathrm{DSE}}{I_{\mathrm{total}}}\right)=\sum_{k=1}^{\infty }\frac{1}{k}{\left(\frac{I_{\mathrm{scatter}},\mathrm{DSE}}{I_{\mathrm{total}}}\right)}^{k}\\ \approx \sum_{k=1}^{K}\frac{1}{k}{\left(\frac{I_{\mathrm{scatter}},\mathrm{DSE}}{I_{\mathrm{total}}}\right)}^{k}. \end{array}$$

In this work we use K=5 to approximate the correction term.

We implemented and tested DSE for single‐source and multi‐source simulations. We use two implementations for single‐source correction:

1. 1‐view DSE: The 1‐view DSE takes one projection as input and predicts the corresponding scatter distribution.
2. 3‐view DSE: The 3‐view DSE has three input channels: One is the projection which we wish to predict the scatter distribution for. The other two are projections with an angular offset of $\pm 10^{\circ }$ to the central projection. The idea behind this implementation is that the additional projections may improve DSE performance since it provides 3D information about the object. We chose an angular offset of 10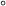 since it was found to yield good results.^28^

As a reference to compare single‐source DSE against, we implemented a kernel‐based method which is a modified version of the model proposed by Ohnesorge et al.^12^ We optimized the kernels with the training dataset using a simplex^39^ algorithm, minimizing the very same loss function (1).

DSE for multi‐source simulations is equivalent to the 1‐view DSE except that the input projection contains the sum of primary and scatter from simultaneously active sources instead of primary and scatter from only one source.

### 2.3 Multi‐source simulations

Since static CT has a ring source array, it is possible to operate this scanner not only in single‐source mode, but also in multi‐source mode. The number of sources that can be fired simultaneously is limited by the field of measurement and the scanner geometry. When firing a certain amount of sources, the primary fan beams of the individual sources will overlap and make the measured data unsuitable or even unusable. We conducted simulations in dual‐source and triple‐source mode. In both modes, sources are operated at a 120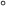 offset. The multi‐source operation leads to the projection data being contaminated with cross‐scatter additionally to the forward scatter. The contaminated projections are obtained by adding the smoothed MC‐estimates of simultaneously active sources.

The DSE architecture remains unchanged for multi‐source correction. However, instead of predicting forward‐scatter only, DSE now predicts the sum of forward‐scatter and cross‐scatter. DSE benefits from the fact that the primary fan beams in multi‐source mode do not overlap, leaving regions on the ring detector that measure only cross‐scatter. These measurements may enable more accurate predictions, as DSE can use them to interpolate between the measured regions and exploit spatial information they provide.

It may be possible to enhance temporal resolution by a factor equal to the number of active sources. However, this comes at the cost of increased noise due to the additional scattered radiation.^40^ For multi‐source operation, we expect high SPRs, meaning that scatter will contribute substantially to image noise. To assess this effect, we evaluate noise levels for an anthropomorphic abdomen phantom and for cylindrical and elliptical water phantoms with diameters of 16 cm, 32 cm, and 30×40 cm. Noisy data are simulated by adding Poisson noise to the intensities in projection domain. The following cases are simulated:

1. N(P): Case without scatter = ground truth (GT). The operator N adds Poisson noise to the analytically calculated scatter‐free projection data P. This case can be seen as some sort of 3rd generation CT reference that is equipped with a very effective ASG.
2. N(P+S): Uncorrected case. Poisson noise is added to the overlay of analytically calculated primary data and smoothed MC scatter estimates S. This case is simulated for operation with single‐source, dual‐source and triple‐source mode.
3. $N\left(\text{P+S}\right)-S_{\text{DSE}}$: DSE‐corrected case. Poisson noise is added as in the uncorrected case. After noise is added, the DSE scatter estimate is subtracted in projection domain.
4. N(P+S)−S: Case with ideal correction. Poisson noise is added as in the uncorrected case. After noise is added, the smoothed MC scatter estimate is subtracted in projection domain. The obtained data are entirely scatter‐free since the MC estimate is the ground truth. However, the quantum noise that was added due to scatter is still present. This case represents the optimum that is achievable with software‐based scatter correction.

We consider the GT case without scatter, corresponding to an ideal or highly effective ASG, as the reference. Then it is possible to calculate the increase in dose for single‐ and multi‐source mode that is necessary to obtain images with the same noise level. This dose penalty can be calculated according to (2), with σ being the image noise level:

(2) $$\text{DosePenalty}=\frac{\sigma_{\text{IdealCorrection}}^{2}}{\sigma_{\text{WithoutScatter}}^{2}}-1$$

The dose penalty can thus be used to evaluate the feasibility of multi‐source operation, as a high dose penalty indicates that longer scan times per projection may be required to achieve comparable image quality.

We further evaluate how scatter affects the contrast‐to‐noise ratio (CNR), calculated between two regions of interest (ROIs) according to (3):

(3) $$\text{CNR}=\frac{|\mu_{1}-\mu_{2}|}{\sqrt{\sigma_{1}^{2}+\sigma_{2}^{2}}}$$

where μ and σ denote the mean CT value and the standard deviation, respectively.

### 2.4 ASG simulations

Inherently, the usage of ASGs for static CT like they are used in 3rd generation CT is not feasible since the ASG cannot follow the changing focus point. However, the absence of an ASG has two major implications: First, it results in a higher SPR at the detector, thereby increasing scatter‐induced artifacts. These artifacts can be effectively corrected for using robust software‐based scatter correction. Second, the increased scattered radiation introduces additional quantum noise, which cannot be removed by software‐based scatter correction and therefore leads to higher image noise. In static CT, classical ASGs with lamellae in *z*‐ and β‐direction would lead to significant absorption of primary radiation at large fan angles. However, most of this absorption is caused by the lamellae in β‐direction. Therefore, we chose to simulate ASGs in *z*‐direction (further called *z*‐ASG) to assess their possible benefits. Figure 4 shows an outline of the simulated *z*‐ASG in the *x*‐*z*‐plane.

**FIGURE 4:**
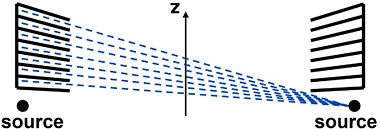
Schematic outline of an ASG in *z*‐direction with eight rows shown for illustration. Dimensions are not drawn to scale. The lamellae of the ASG are adjusted in each row so that they align with different points along the *z*‐axis, allowing the grid to focus on the source opposite the detector element.

The ASG for a given detector channel can be aligned in three dimensions only to one point that lies exactly opposite the channel. This holds also for an ASG with lamellae in *z*‐direction only. For focus positions that deviate from this exactly opposite point, this alignment is then only approximate, which results in partial shadowing of the primary radiation from these focus locations in the given channel. This shadowing increases continuously the further away the focus is from the exact opposite point. From the point of view of the focus, this means that for a given fan beam emanating from a single focus, the primary rays within that beam are increasingly shadowed towards the edge of the measurement field. With a *z*‐ASG and as the FoM is not used to its maximum, the net effect of blocking scattered radiation on the one hand and shadowing primary on the other hand can still be beneficial for the resulting signal‐to‐noise ratio in the reconstructed images of the measured data.

We simulated *z*‐ASGs with a height ranging from 5 to 40 mm. Lamellae of the *z*‐ASGs are assumed to possess an infinite absorption coefficient, resulting in complete absorption of photons striking them. Moreover, we assume that the lamellae overlap perfectly with the optical separators of the detector pixels. Consequently, no primary rays that would otherwise contribute to imaging are blocked due to the thickness of the lamellae. We simulated tube voltages ranging from 70 to 140 kV. Although the maximum transmission angle of an ASG is independent of the tube voltage, the tube voltage impacts the SPR at the detector and thereby ASG performance. Electronic noise of the detector is neglected since modern detectors exhibit low readout noise, leading to image noise being mainly determined by quantum noise for typical dose levels and patient sizes.^41^ This also holds for larger angles where the *z*‐ASG causes partial shadowing of the primary signal since the thickness of the scanned object typically decreases off‐center, resulting in a higher primary signal at the detector. However, electronic noise may be relevant in very low‐dose scan protocols, which we do not consider in this work. Two scanner geometries were simulated: one with a detector *z*‐coverage of 12 mm at the isocenter (16 rows) and another with a detector *z*‐coverage of 48 mm (64 rows). Simulation settings are summarized in Table 4.

**TABLE 4:** Specifications of the *z*‐ASG simulations.

Tube voltage	70–140 kV
ASG heights	0 mm, 5 mm, 20 mm, 40 mm
Detector *z*‐coverage (isocenter)	12 mm, 48 mm
Lamellae thickness	Negligible / Overlaps with optical separators
Electronic noise	Neglected
Phantom diameter	16 cm, 32 cm, 40 cm

As a figure of merit we calculate the signal‐to‐noise ratio (SNR) improvement factor (SNR_if_), which is defined by Equation (4):

(4) $${\mathrm{SNR}}_{\text{if}}=\frac{{\mathrm{SNR}}_{\text{WithASG}}}{{\mathrm{SNR}}_{\text{WithoutASG}}}$$

with the SNR being calculated according to Equation (5):

(5) $$\mathrm{SNR}=\frac{T_{P}I_{P}}{\sqrt{T_{P}I_{P}+T_{S}I_{S}}}.$$

*T*_P_, *T*_S_ are the primary and scatter transmission and *I*_P_, *I*_S_ are the primary and scatter intensities, respectively. A derivation of (5) can be found in the Appendix A.1. Since *T*_P_, *T*_S_, *I*_P_, and *I*_S_ are functions of the angle within the primary fan, the resulting SNR_if_ is as well. ${\mathrm{SNR}}_{\text{if}}>1$ means that static CT benefits from using the simulated *z*‐ASG whereas ${\mathrm{SNR}}_{\text{if}}<1$ corresponds to a decrease in SNR.

## 3 RESULTS

### 3.1 Single‐source simulations

Figure 5 shows scatter‐corrected images of an abdomen and a head phantom.

**FIGURE 5:**
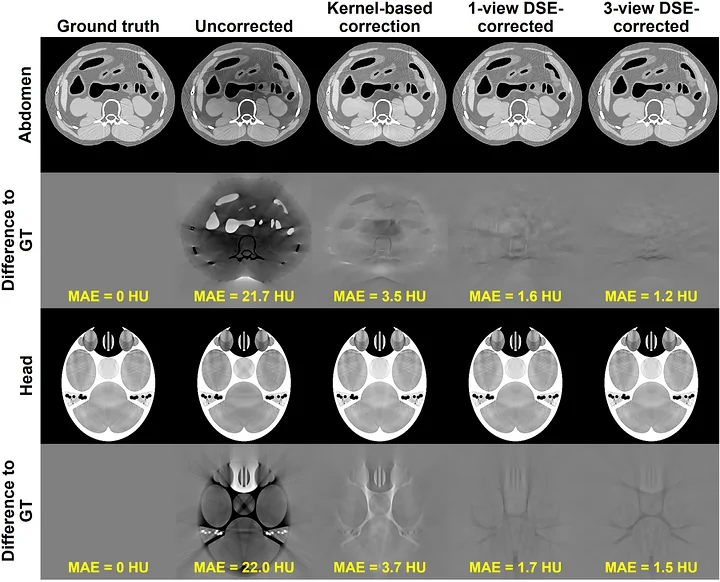
Scatter correction of an abdomen and a head phantom. Severe scatter artifacts are visible in the uncorrected reconstruction, resulting mainly as cupping in the image center and streak artifacts. Both, 1‐view DSE and 3‐view DSE, perform very well and outperform the kernel‐based reference method. 120 kV, 0 mm *z*‐ASG, 12 mm detector. Reconstructions: *C* = 0 HU, *W* = 300 HU. Difference images: *C* = 0 HU, *W* = 100 HU.

The uncorrected reconstruction of the abdomen phantom results in strong scatter artifacts, appearing mainly as cupping and some streaks between regions of high attenuation. For quantitative assessment, the mean absolute error (MAE) was calculated over the entire phantom, which is 21.7 HU. Both, 1‐view DSE and 3‐view DSE perform very well, reducing the MAEs to 1.6 HU and 1.2 HU, respectively, with no visible scatter artifacts remaining in the reconstructions. The kernel‐based method also performs very well with a slightly higher remaining MAE of 3.5 HU.

The head phantom is positioned 8 cm off‐center. The uncorrected reconstruction yields an MAE of 22.0 HU, which is reduced to 1.7 HU with 1‐view DSE and 1.5 HU with 3‐view DSE. Again, the kernel‐based method produces the highest MAE of 3.7 HU and results in slight overcorrection. Figure 6 shows the XCAT chest phantom with contrast agent. 1‐view DSE and 3‐view DSE reduce the MAE from 32.5 to 4.5 HU and 2.1 HU, respectively. The uncorrected image suffers from severe cupping and streak artifacts. The 1‐view DSE approach substantially reduces these artifacts, but leaves minor residual shading in the vicinity of the heart. In contrast, no visible artifacts remain after correction with 3‐view DSE. The kernel‐based method reduces the MAE to 9.2 HU. However, the corrected image shows slight undercorrection in the object center and a severe streak artifact at the spine caused by overcorrection.

**FIGURE 6:**
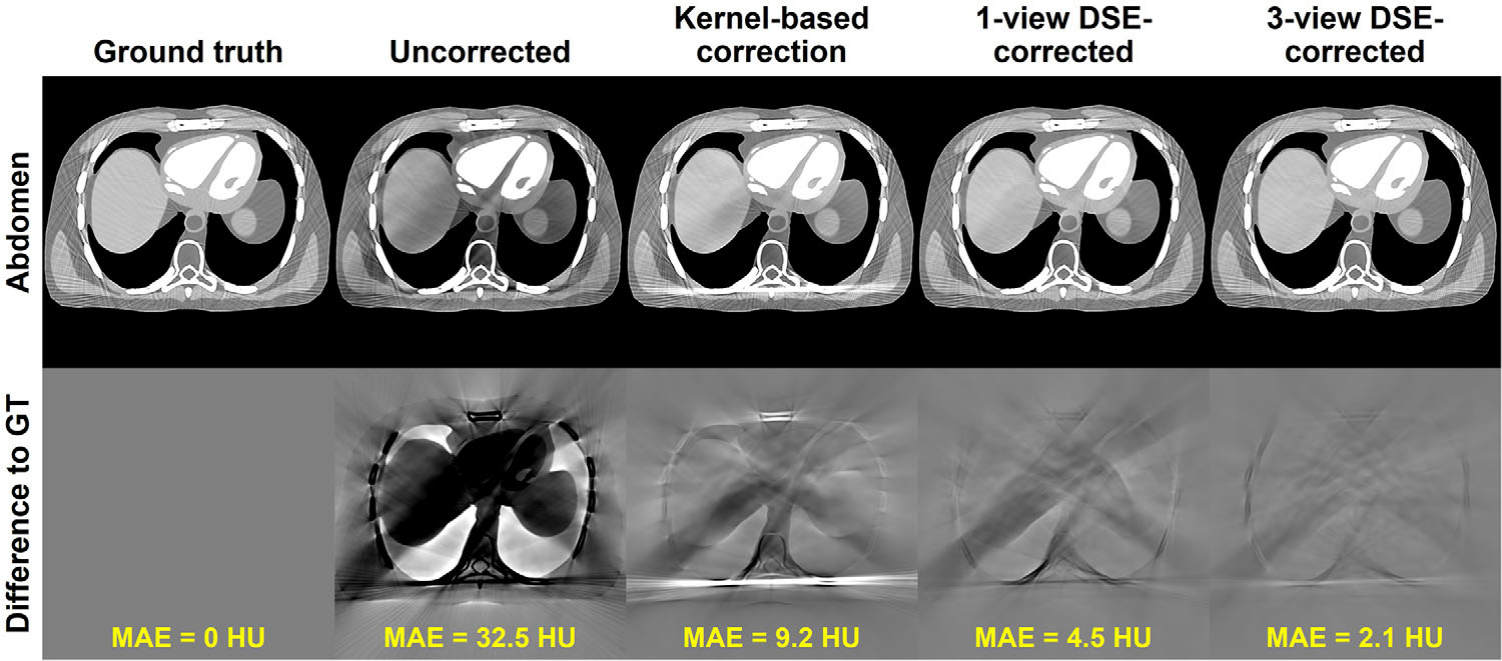
DSE applied to an XCAT chest phantom with contrast agent. The 1‐view DSE approach shows minor residual shading near the heart, 3‐view DSE completely removes visible artifacts. The kernel‐based approach introduces a pronounced streak artifact at the spine. 120 kV, 0 mm *z*‐ASG, 12 mm detector. Reconstructions: *C* = 0 HU, *W* = 200 HU. Difference images: *C* = 0 HU, *W* = 100 HU.

### 3.2 Multi‐source simulations

Figure 7 shows reconstructions of the same XCAT abdomen phantom, comparing operation with one, two and three sources simultaneously.

**FIGURE 7:**
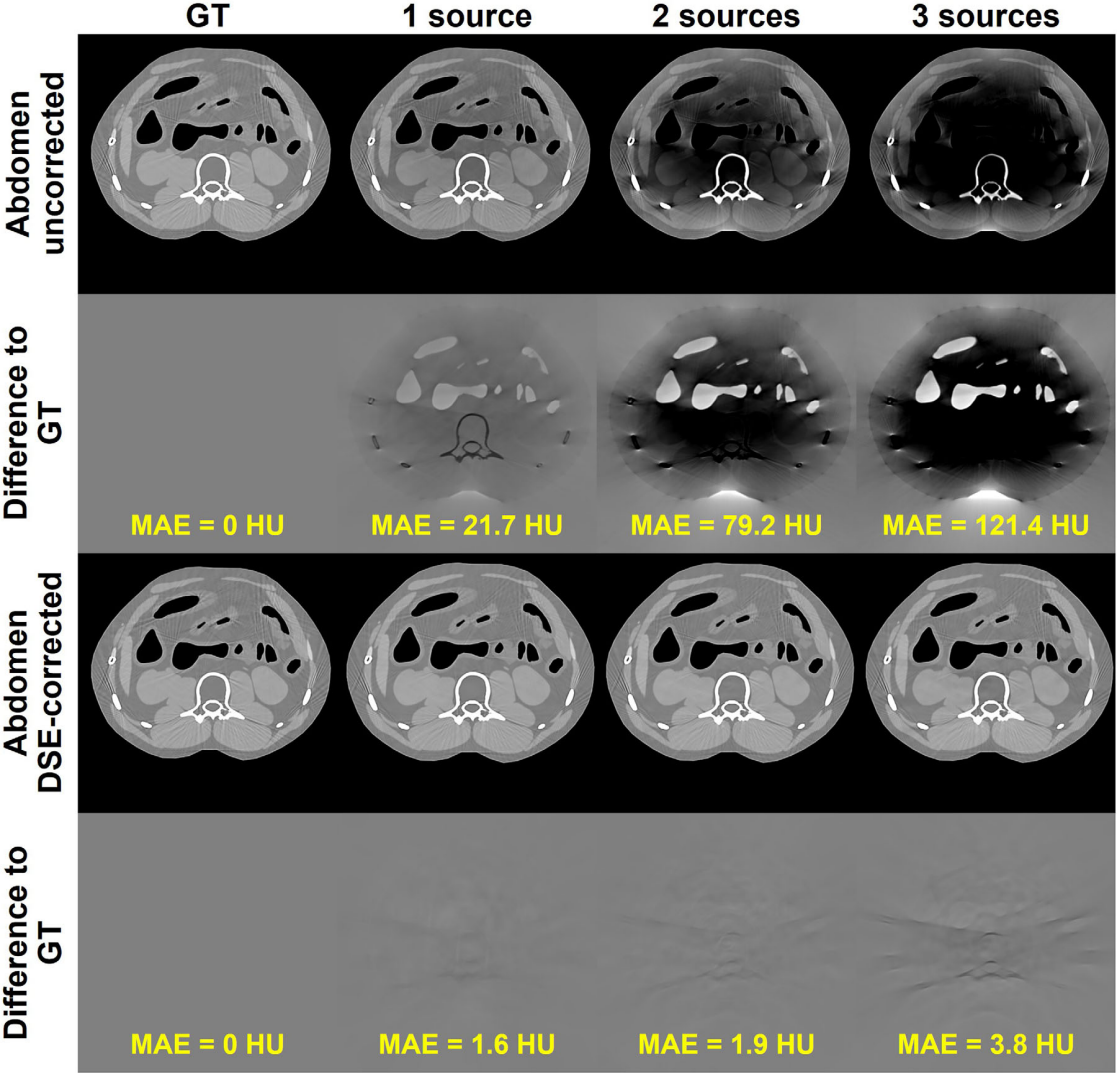
Multi‐source scatter simulations. Operation with more than one source leads to a substantial increase in artifacts. DSE is able to correct the cross‐scatter artifacts very well. 120 kV, 0 mm *z*‐ASG, 12 mm detector. Reconstructions and difference images: *C* = 0 HU, *W* = 300 HU.

Without scattered radiation correction, severe scatter artifacts are observed, particularly in dual‐ and triple‐source modes. These artifacts manifest as cupping over the phantom and streaks between bones. The MAE over the entire phantom is 21.7, 79.2, and 121.4 HU for one, two and three sources, respectively. DSE is able to reduce these MAEs to 1.6, 1.9, and 3.8 HU. Figure 8 shows performance of multi‐source DSE for a chest phantom with contrast agent. Although training data did not include any phantoms with contrast agent, DSE performance holds with a reduction of the MAE from 113.7 to 7.1 HU for dual‐source mode, and from 164.1 to 12.4 HU for triple‐source mode. Scatter artifacts are strongly reduced, with a slight streak artifact remaining visible in the spinal region.

**FIGURE 8:**
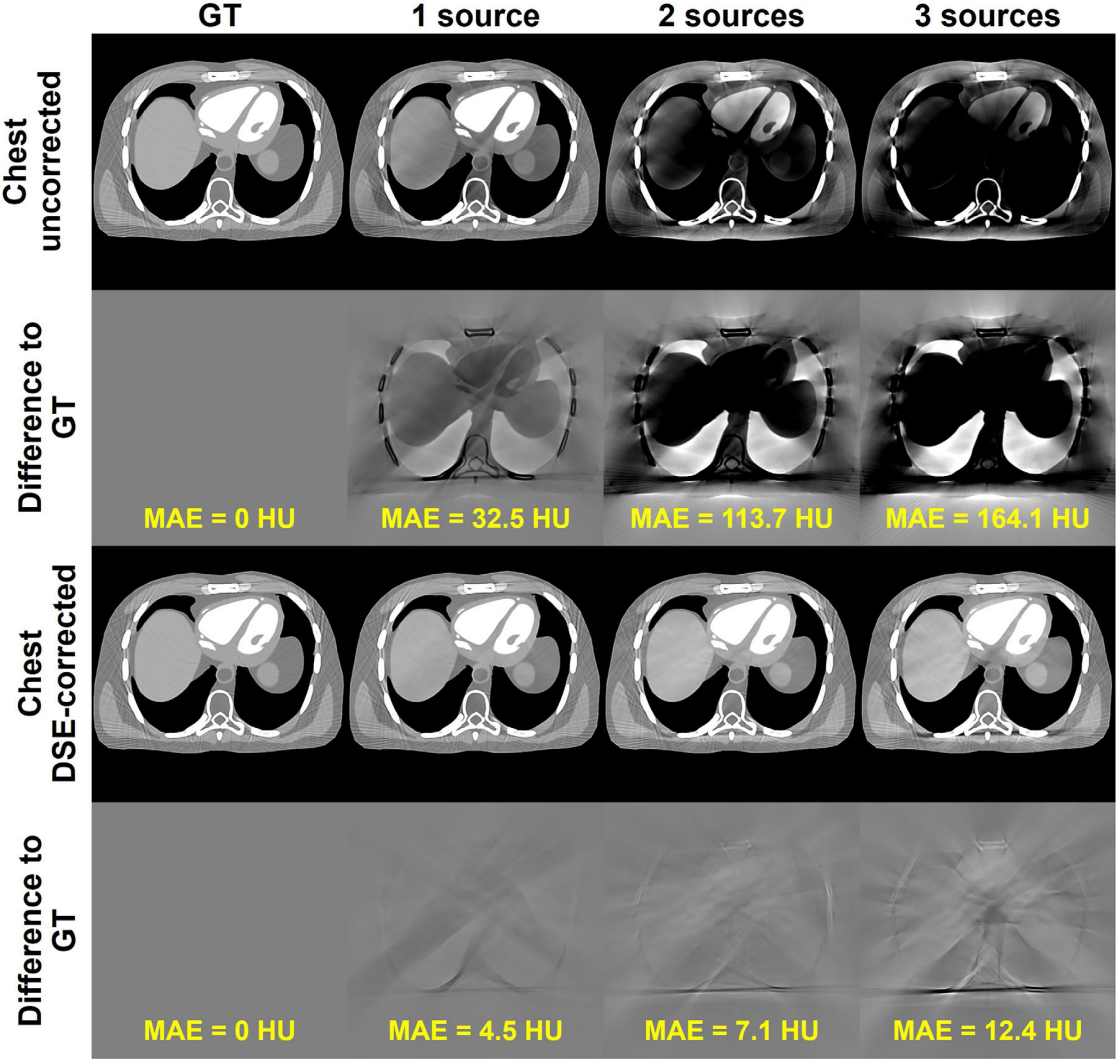
Multi‐source scatter simulations of a chest phantom. Performance of DSE holds also when applied to phantoms with contrast agent. A slight streak artifact remains for triple‐source operation. 120 kV, 0 mm *z*‐ASG, 12 mm detector. Reconstructions and difference images: *C* = 0 HU, *W* = 300 HU.

Figure 9 shows noise simulations for the abdomen phantom. We compare two scatter correction methods: Correction with DSE and ideal correction, the latter referring to correction using the smoothed MC estimate. The image pixel noise is approximately set equal to the standard deviation calculated from the CT values within ROI_1_ and ROI_2_ shown in Figure 9, assuming that phantom structures or deterministic variations of CT values due to scattered radiation (e.g., cupping) across these ROIs can be neglected.

**FIGURE 9:**
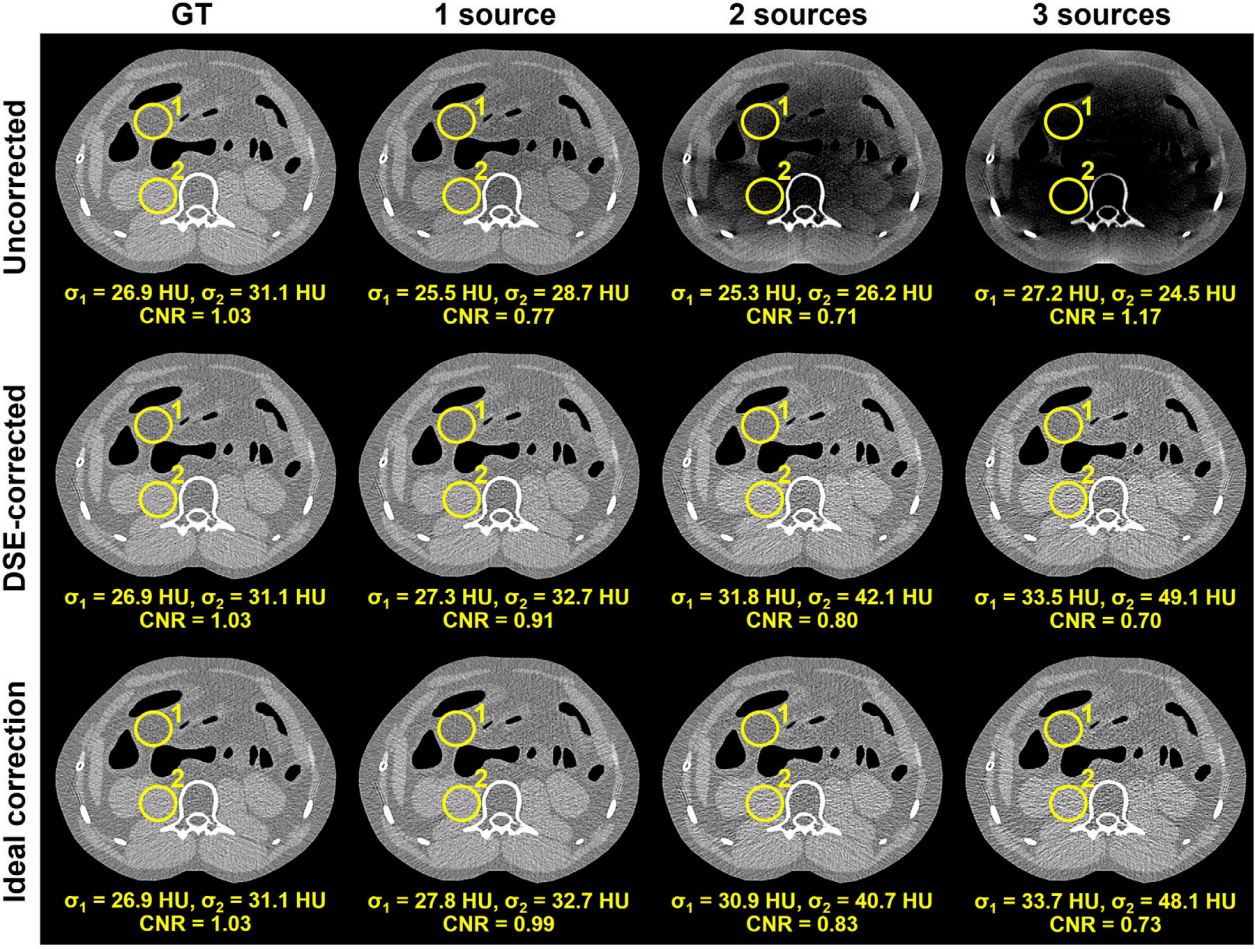
Multi‐source noise simulations with an anthropomorphic XCAT phantom. $\sigma_{1}$ and $\sigma_{2}$ correspond to the noise levels in ROI_1_ and ROI_2_, measured as the standard deviation of the CT value. All images were simulated at the same dose, with the noise level selected to reflect a realistic scan. 120 kV, 0 mm *z*‐ASG, 12 mm detector. Reconstruction window: *C* = 0 HU, *W* = 300 HU.

The noise level increases in all corrected images compared to the GT, with the increase depending on the evaluated region. For operation with three sources, the noise level in ROI_1_ increases from 26.9 to 33.7 HU (≈25%), while ROI_2_ shows a larger increase from 31.1 to 48.1 HU (≈55%). Furthermore, the CNR decreases in the uncorrected images except for operation with three sources, where CNR appears to be increased from 1.03 to 1.17. Scatter correction partially restores the CNR. However, the CNR of the GT cannot be fully recovered, even with ideal correction. Figure 10 presents noise simulations for simple water phantoms acquired with one, two, and three sources, allowing the dependence of image noise on phantom size to be evaluated.

**FIGURE 10:**
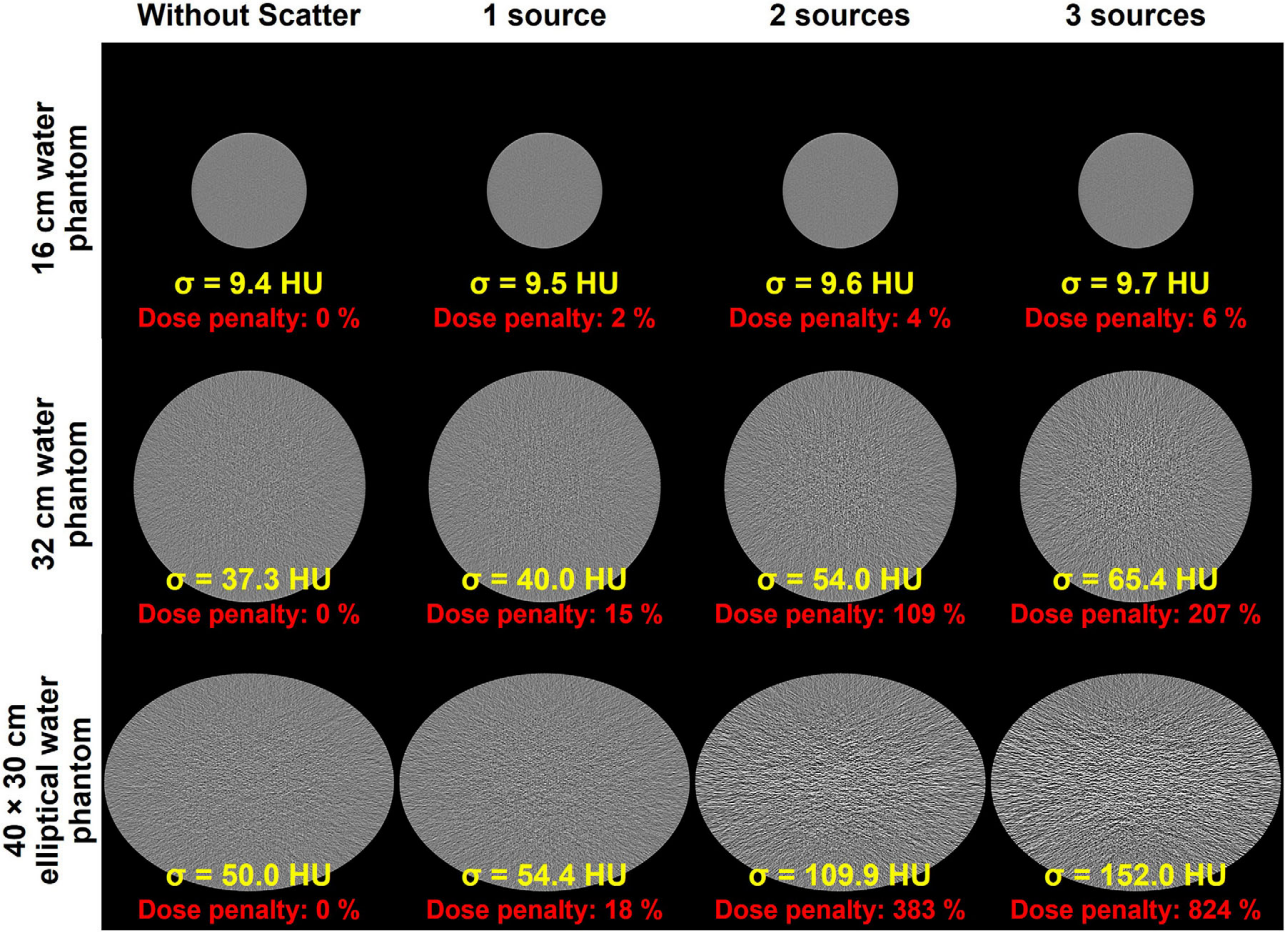
Noise in simulated images without scattered radiation (first column) and scattered radiation corrected images with one, two and three sources (second, third, and fourth column, respectively). Noise simulations were performed with water cylinders of 16 and 32 cm diameter, as well as an elliptical water phantom measuring 30×40cm. All simulations were conducted at a constant dose for the three phantoms. The increase in noise is strongly dependent on phantom size: while the 16 cm phantom shows only a negligible increase, a substantial increase is observed for the 32 cm phantom and the 30×40cm phantom. To obtain a comparable noise level with multi‐source operation as with single‐source operation, the dose would need to be increased accordingly, resulting in a dose penalty. 120 kV, 0 mm *z*‐ASG, 12 mm detector. Reconstruction window: *C* = 0 HU, *W* = 500 HU.

The noise level was measured as the standard deviation over the entire phantom. Ideal software‐based scatter correction was assumed, that is, correction with the simulated GT. Therefore, no systematic deviations of CT values should exist that impair the noise measurement. For the 16 cm water phantom, the noise level in a simulation where scattered radiation was not registered is 9.4 HU. With the scatter‐corrected images it increases only slightly to 9.5 and 9.6 HU and 9.7 HU for one and two and three sources, respectively. The increase in noise is more pronounced for the 32 cm water phantom, where noise increases from 37.3 HU without scatter to 40.0, 54.0, and 65.4 HU for one, two, and three sources, respectively. The largest increase occurs for the 30×40cm elliptical phantom, from 50.0 HU without scatter to 54.4, 109.9, and 152.0 HU for one, two, and three active sources, respectively. Based on Poisson statistics, the dose increase required to achieve image quality equivalent to that of the scatter‐free reconstruction is calculated using (2). For the 16 cm phantom this increase is 2 %, 4 % and 6 % for one, two and three sources. For the 32 cm water phantom the increase is 15 %, 109 %, and 207 %, and for the 30×40cm phantom it is 18 %, 383 %, and 824 % for operation with one, two and three sources, respectively.

### 3.3 ASG simulations

Figure 11 illustrates the SPR for 12 and 48 mm detectors, simulated for a 32 cm cylindrical water phantom and *z*‐ASGs of 5, 20, and 40 mm height.

**FIGURE 11:**
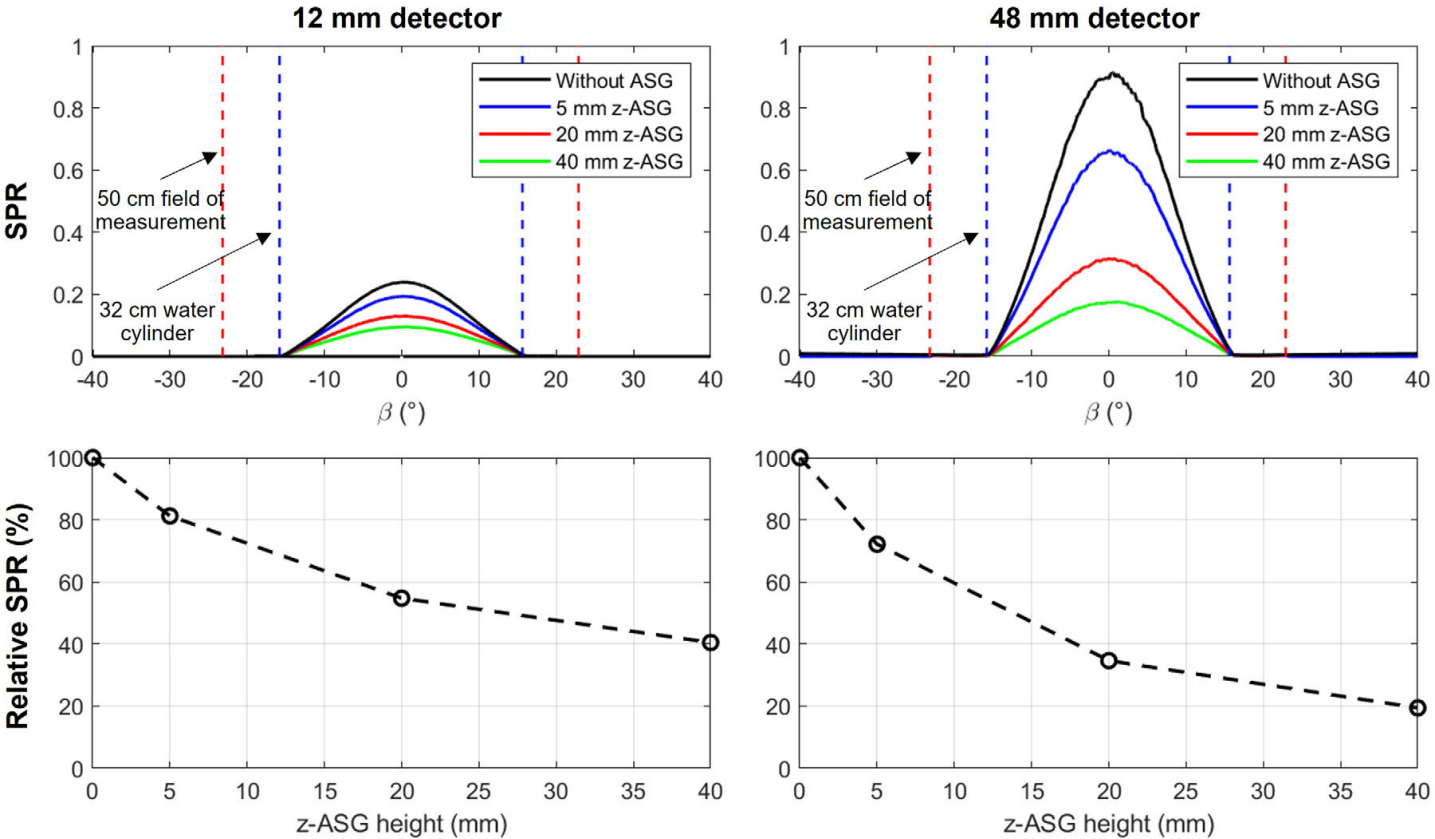
SPR as a function of the fan angle (upper row) and relative SPR as a function of *z*‐ASG height (lower row), evaluated for a 32 cm water phantom. 120 kV, 0 to 40 mm *z*‐ASG, 12 and 48 mm detectors.

The SPR was averaged over all detector rows. In the absence of a *z*‐ASG, the maximum SPR reaches approximately 0.24 and 0.94 for the 12 and 48 mm detectors, respectively. The relative SPR quantifies the reduction of the SPR achieved by the *z*‐ASG. The steepest decrease is observed at small *z*‐ASG heights: A 5 mm *z*‐ASG reduces the SPR to 80 %, while increasing the height to 20 mm further lowers the SPR to below 60 % for the 12 mm detector. The effect of *z*‐ASGs is even more pronounced for the 48 mm detector, where a 20 mm *z*‐ASG reduces the SPR to below 40 %. In the following, SNR_if_ is evaluated for a *z*‐ASG height of 20 mm. This height was selected because it is considered feasible from a manufacturing perspective and it is within the range of ASG heights employed in existing clinical CT systems. Figure 12 shows the SNR_if_ for a 32 cm water phantom with 12 and 48 mm detectors.

**FIGURE 12:**
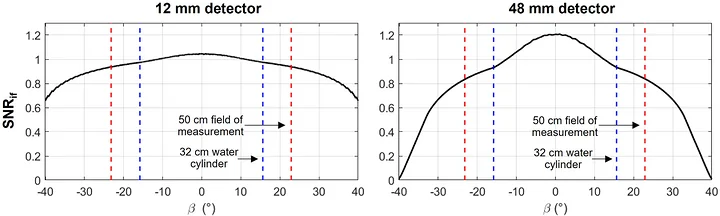
SNR_if_ as a function of the fan angle, evaluated for a 32 cm water phantom. The SNR_if_ falls below unity because the *z*‐ASG becomes increasingly misaligned, resulting in absorption of primary rays. 120 kV, 20 mm *z*‐ASG, 12 and 48 mm detectors.

The maximum SNR_if_ is approximately 1.05 for 12 mm and about 1.20 for 48 mm. The SNR_if_ decreases with increasing fan angle and falls below unity within the boundaries of the water phantom. The region outside the phantom is not relevant since it does not contribute to the reconstruction. The mean SNR_if_ within the phantom boundaries is approximately 1.016 for a *z*‐coverage of 12 mm and 1.094 for a *z*‐coverage of 48 mm. As shown in Figure 13, SPR and SNR_if_ exhibit a dependence on tube voltage, with lower values observed at higher tube voltages. The lowest SPRs were observed for the 16 cm water phantom with 20 mm ASG, ranging from 0.049 at 70 kV to 0.035 at 140 kV. For the 40 cm water phantom, SPRs of 0.57 and 0.42 were observed at 70 and 140 kV, respectively. These changes correspond to relative reductions of SPR by 29 % and 26 %, respectively, resulting from the increased tube voltage. For the 20 mm *z*‐ASG, SNR_if_ decreased slightly from 1.004 at 70 kV to 1.003 at 140 kV for the 16 cm water phantom, and from 1.135 to 1.098 for the 40 cm water phantom.

**FIGURE 13:**
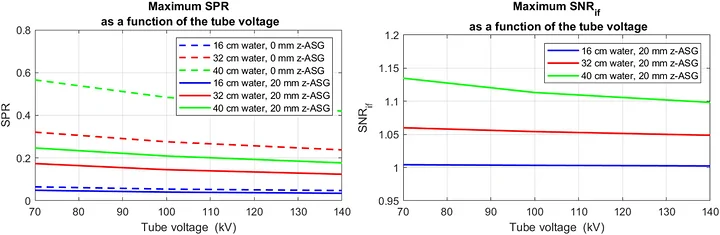
Tube voltage dependency of the SPR and SNR_if_, evaluated for 16, 32, and 40 cm water. Both, SPR and SNR_if_ show a tube voltage dependency with values decreasing for higher tube voltages. 70 to 140 kV, 0 and 20 mm *z*‐ASG, 12 mm detector.

Figure 14 shows how the SNR_if_ translates into image domain. It shows the noise level for a 32 cm water phantom without an ASG, with a 5 mm *z*‐ASG and with a 20 mm *z*‐ASG. Reconstructions are compared for scenarios without scatter, with scatter, and for scatter‐corrected images. As before, we assume ideal scatter correction, realized by correcting with the simulated GT.

**FIGURE 14:**
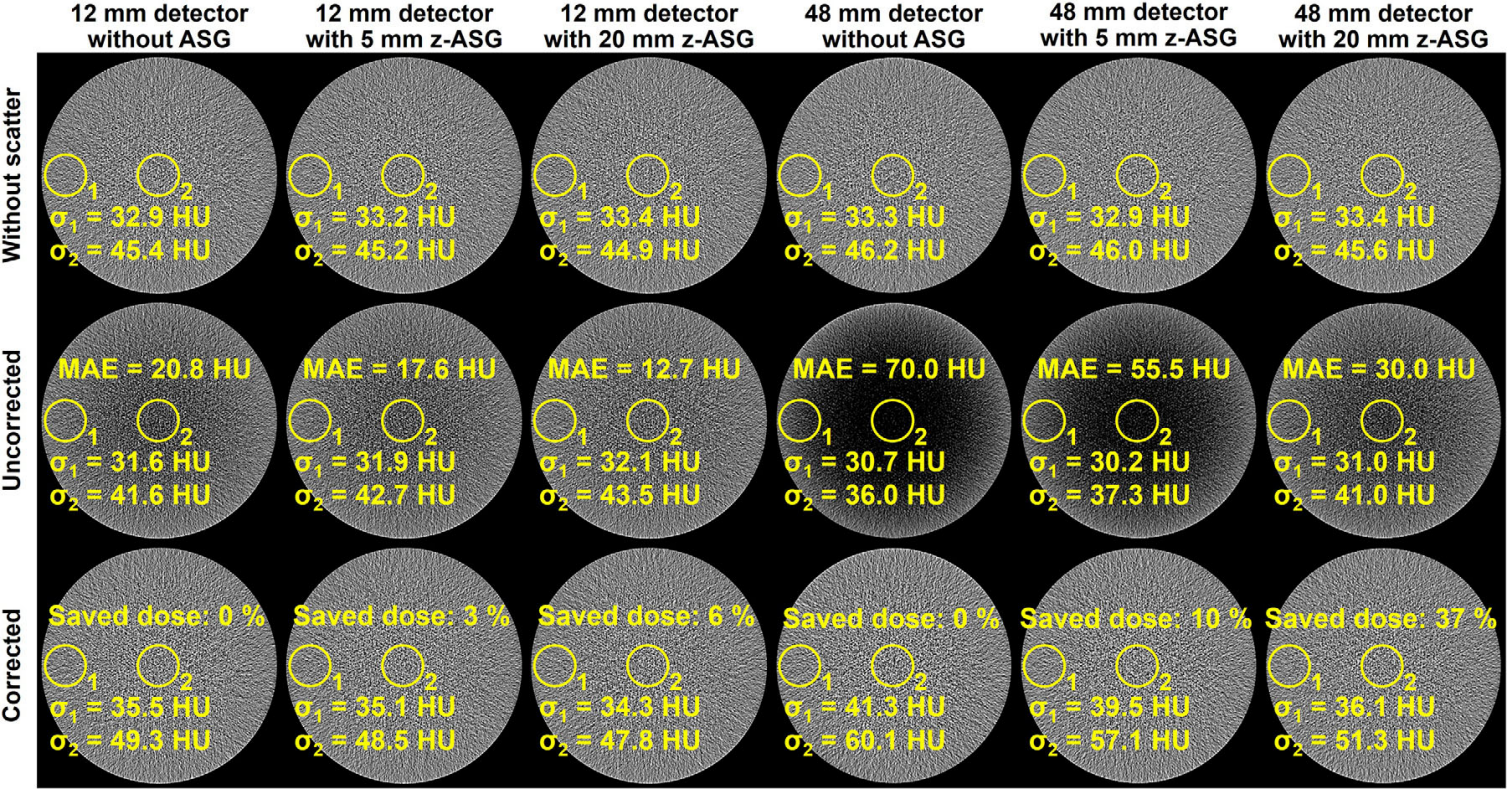
Reconstructions of a 32 cm water phantom without an ASG, with a 5 mm *z*‐ASG, and with 20 mm *z*‐ASG. Scatter artifacts in the image are reduced when *z*‐ASGs are used. However, noise increases in the scatter‐corrected case compared to reconstructions without scatter. The increase in noise depends on *z*‐ASG height and allows to compare the dose that is necessary to achieve equivalent image quality. Scatter corrections were performed assuming ideal software‐based correction. Reconstructions: *C* = 0 HU, *W* = 200 HU. 120 kV, 0 to 20 mm *z*‐ASG, 12 and 48 mm detector.

## 4 DISCUSSION

### 4.1 Single‐source simulations

We compared a 1‐view DSE and a 3‐view DSE with a kernel‐based reference method. Reconstructions were shown for a semi‐anthropomorphic head phantom as well as anthropomorphic abdomen and chest phantoms. The test scenarios thereby cover application in head CT, for example, as a compact and mobile scanner for stroke imaging, and chest CT with contrast agent, an application where temporal resolution is critical due to cardiac movement. Although DSE was trained only on thorax and water phantoms, it consistently outperformed the reference method on the test phantoms, indicating that it generalizes very well to out‐of‐distribution data. Performance also holds in the presence of iodinated contrast media with a CT‐value offset of 300 HU compared to water. DSE effectively corrected for scatter, with no visible scatter artifacts remaining in the 3‐view DSE images and only minor residual shading observed for 1‐view DSE. The thorax phantom used for training is defined as a constructive solid geometry, using simple geometrical shapes to assemble a more complex phantom. Nevertheless, results show convincing performance on the anthropomorphic XCAT phantoms, which are derived from real patient scans and allow arbitrary shapes of the anatomy. This indicates that DSE may perform also well on real patient data. It was also demonstrated by Hennemann et al. that DSE trained on phantoms can be applied to real photon‐counting CT measurements.^30^ We expect that DSE performance may be further improved by enhancing the training dataset by more complex and application‐specific data. To be applied in a physical scanner, DSE would need to be trained and tested for a range of acquisition protocols that are representative of the specific use case. These results indicate that deep learning‐based methods such as DSE have the potential to overcome challenges associated with scatter artifacts in single‐source static CT.

### 4.2 Multi‐source simulations

We simulated anthropomorphic abdomen and chest phantoms operated with one, two and three sources simultaneously. Operation with two and three sources increased the MAE from 21.7 to 79.2 HU and 121.4 HU for the abdomen phantom and from 32.5 to 113.7 HU and 164.1 HU for the chest phantom. Compared to clinical single‐ or dual‐source scanners, artifacts are particularly severe due to the absence of an ASG. Multi‐source operation leads to particularly large MAEs because of cross‐scatter. Nevertheless, DSE corrects cross‐scatter effectively. This may be because DSE has access to an entire detector ring of measured data, including regions between adjacent primary fan beams where only cross‐scatter is measured. DSE appears capable to interpolate very well between these regions. As a result, the remaining MAE of corrected images for two and three active sources is drastically reduced. These findings demonstrate that scatter artifacts for static CT, even with multi‐source operation, can be corrected effectively using DSE. However, our noise simulations show that this leads to a substantial increase in image noise. For example, for dual‐source mode, the dose penalty compared to single‐source mode is 82 % for the 32 cm water phantom. Depending on the use case, these findings may have various implications: First, to maintain the same noise level, static CT in dual‐source mode requires a 1.82‐fold higher dose than in single‐source mode. Second, if applying the same dose, then operating in multi‐source mode will yield enhanced temporal resolution at the expense of substantially increased image noise. Third, assuming that the sources are operated at maximum source power and that a certain noise level must not be exceeded, then multi‐source operation may loose its advantage regarding temporal resolution. For dual‐source operation, this is the case when the dose penalty exceeds 100 % since in this case double the dose needs to be applied to achieve the desired noise level. At maximum source power, this equals to doubled acquisition time per projection. Our results indicate that the dose penalty is strongly dependent on object size, ranging for operation with three sources from 6 % (16 cm water phantom) to 824 % (30×40cm water phantom). Therefore, we conclude that multi‐source operation in static CT may be advantageous for small objects when high temporal resolution is critical, but for larger objects, it primarily results in unnecessary dose.

Evaluating noise levels in the test abdomen phantom reveals that the increase in noise also depends on the ROI. In triple‐source mode, the dose penalty is 57 % for ROI_1_, but 139 % for ROI_2_, demonstrating that not only phantom size but also the specific region within the phantom or patient is crucial when assessing the benefit of operating with multiple sources. The CNR cannot be fully recovered even with optimal software‐based correction, as noise increases. Therefore, multi‐source operation at the same dose will always lead to lower CNR compare to single‐source operation. It may be noticed that, in our test case (Figure 9), triple‐source operation leads to increased CNR in the scatter‐corrupted image. Such an increase may occur for two reasons. First, scatter‐induced cupping causes a deterministic alteration of the CT values within the ROIs used for CNR measurement, thereby either increasing or decreasing the contrast. Second, the scattered radiation enhances photon statistics and thus decreases noise in the uncorrected measurements. In the present case, the contrast between the two ROIs is exactly inverted from 43 to −43 HU due to cupping and, thus, the reduced noise leads to increased CNR. This does not represent a real improvement in CNR, but is a result of artifacts. As expected, once scatter is corrected, the CNR drops below the respective values of single‐source and dual‐source operation.

### 4.3 ASG simulations

We simulated ASGs in the z‐direction, with heights of 5, 20 and 40 mm. Simulations were conducted for 12 and 48 mm detectors in single‐source mode, and scatter correction was performed assuming ideal software‐based correction, that is, with the simulated smoothed MC estimates. All investigated *z*‐ASGs effectively reduced SPR and, consequently, scatter‐related artifacts, irrespective of phantom size and tube voltage. *z*‐ASGs were more effective for the 48 mm detector since this configuration uses a wider collimation in the *z*‐direction, allowing scattered radiation to reach the detector from a larger *z*‐range. However, despite effectively reducing SPR, employing a *z*‐ASG may still result in a reduced SNR. This occurs because, at larger angles within the fan, slightly misaligned lamellae block part of the primary signal. Since SNR is a key metric for assessing image quality in terms of noise, SNR_if_ was evaluated across tube voltages and object sizes. For a 32 cm water cylinder and 20 mm *z*‐ASG, our results show that SNR_if_ at fan angle 0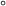 (i.e., at the detector element opposite to the active source) exceeds 1, indicating that the *z*‐ASG is beneficial. This makes sense since the *z*‐ASG is well aligned at that point and does not block primary rays. Here as well, *z*‐ASG is more effective for the 48 mm detector. However, SNR_if_ falls below unity within the phantom boundaries, suggesting that the *z*‐ASG degrades SNR. Therefore, the overall benefit of the *z*‐ASG for image quality cannot be conclusively assessed based solely on SNR_if_. Figure 14 displays reconstructions using *z*‐ASGs with heights of 5 and 20 mm for a 32 cm water phantom. As expected, scatter artifacts decrease with increasing *z*‐ASG height, consistent with the relative SPR shown in Figure 11. Interestingly, image noise is lower not only in the central region of the phantom but also at its edges, even though the SNR_if_ dropped below 1 at larger angles. This is possible because the reconstructed attenuation values are result from backprojection of the line integrals spanning 180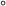, which includes integrals passing through the object center with SNR_if_ greater than 1. These integrals are critical for image noise since x‐ray intensity is highly attenuated in the center, leading to poor photon statistics compared to integrals at the phantom edge. This effect is especially pronounced in the simulated static CT since no bowtie filter was used. Since both the 5 and 20 mm *z*‐ASGs consistently reduce image noise compared to the case without ASG, these results indicate that static CT can benefit from the use of a *z*‐ASG. The dose savings strongly depend on collimation, phantom size and tube voltage. Results from the 32 cm water phantom cannot be directly transferred to phantoms of other sizes. The benefit of *z*‐ASGs seems to be limited for smaller phantoms, whereas it becomes more pronounced for larger phantoms. In this work, we demonstrated the potential benefit for the simulated scanner geometry. However, there is currently no consensus regarding the optimal design of static CT systems, and the observed results may depend on the scanner geometry. In particular, the detector radius and its ratio to the source radius may influence the benefit provided by a *z*‐ASG. Therefore, a sensitivity analysis of the impact of scanner geometry could be an important subject for future work and may contribute to the optimization of static CT system design.

## 5 CONCLUSION

Results of our simulation study demonstrate that scatter artifacts in fourth generation static CT systems can be effectively reduced by applying DSE, not only in single‐source mode but also in multi‐source mode when facing extremely high SPRs. The results indicate how scatter contributes to image noise. Therefore, multi‐source operation may be suitable only in specific use cases where high temporal resolution is critical and the scanned object is relatively small. For a prospective realization of multi‐source static CT, it would be necessary to develop a figure of merit to determine when to operate in single‐source and when to use multi‐source mode. The development of such a figure of merit could be part of future work. Factors to consider may include object attenuation, object size, patient heart rate, and expected SPR. Some of these factors can be easily obtained from a topogram. Although the deployment of a conventional two‐dimensional ASG is not feasible for static CT, our findings indicate that ASGs in the *z*‐direction can successfully reduce image noise and improve SNR and thereby enable dose reductions. They should therefore be considered as an option, particularly when scanning larger objects or designing scanners with larger *z*‐coverage.

## FUNDING

This study was funded by Siemens Healthineers AG, Forchheim, Germany.

## CONFLICT OF INTEREST STATEMENT

Andreas Heinkele, Lukas Hennemann, Julien Erath, Johan Sunnegårdh, Christian Hofmann, Martin Petersilka and Karl Stierstorfer are employees of Siemens Healthineers AG.

## APPENDIX A SNR CALCULATION

Be $I_{P}$ and $I_{S}$ the primary and scatter intensities reaching the ASG, and $T_{P}$ and $T_{S}$ the transmission rate of primary and scatter through the ASG. Then, the primary radiation $I_{P,\det}$ reaching the detector is given by

(A1)
$$I_{P,\det}=T_{P}I_{P}\;.$$

The noise level in projection domain is determined by the total radiation $I_{T,\det}$ reaching the detector, that is, by the overlay of primary and scatter. Since the incoming radiation follows Poisson statistics, we can use its property that the variance $\sigma^{2}$ is equal to its expectation value to obtain

(A2)
$$I_{T,\det}=\sigma^{2}=T_{P}I_{P}+T_{S}I_{S}\;.$$

Combining (A1) and (A2) yields the formula for the SNR:

(A3)
$$\mathrm{SNR}=\frac{I_{P,\det}}{\sigma }=\frac{T_{P}I_{P}}{\sqrt{T_{P}I_{P}+T_{S}I_{S}}}\;.$$

Simulation without ASG corresponds to a special case of (A3) where $T_{P}$ and $T_{S}$ are equal to 1.
